# The Arabinose 5-Phosphate Isomerase KdsD Is Required for Virulence in Burkholderia pseudomallei

**DOI:** 10.1128/jb.00034-23

**Published:** 2023-07-17

**Authors:** Christopher H. Jenkins, Andrew E. Scott, Paul A. O’Neill, Isobel H. Norville, Joann L. Prior, Philip M. Ireland

**Affiliations:** a Chemical, Biological and Radiological Division, Defence Science and Technology Laboratory, Salisbury, Wiltshire, United Kingdom; b University of Exeter Sequencing Service, Exeter, United Kingdom; c Biosciences Department, University of Exeter, Exeter, United Kingdom; d Southampton General Hospital, Southampton, United Kingdom; Geisel School of Medicine at Dartmouth

**Keywords:** *Burkholderia pseudomallei*, lipopolysaccharide, capsule, virulence, arabinose 5-phosphate isomerase

## Abstract

Burkholderia pseudomallei is the causative agent of melioidosis, which is endemic primarily in Southeast Asia and northern Australia but is increasingly being seen in other tropical and subtropical regions of the world. Melioidosis is associated with high morbidity and mortality rates, which is mediated by the wide range of virulence factors encoded by B. pseudomallei. These virulence determinants include surface polysaccharides such as lipopolysaccharide (LPS) and capsular polysaccharides (CPS). Here, we investigated a predicted arabinose-5-phosphate isomerase (API) similar to KdsD in B. pseudomallei strain K96243. KdsD is required for the production of the highly conserved 3-deoxy-d-manno-octulosonic acid (Kdo), a key sugar in the core region of LPS. Recombinant KdsD was expressed and purified, and API activity was determined. Although a putative API paralogue (KpsF) is also predicted to be encoded, the deletion of *kdsD* resulted in growth defects, loss of motility, reduced survival in RAW 264.7 murine macrophages, and attenuation in a BALB/c mouse model of melioidosis. Suppressor mutations were observed during a phenotypic screen for motility, revealing single nucleotide polymorphisms or indels located in the poorly understood CPS type IV cluster. Crucially, suppressor mutations did not result in reversion of attenuation *in vivo*. This study demonstrates the importance of KdsD for B. pseudomallei virulence and highlights further the complex nature of the polysaccharides it produces.

**IMPORTANCE** The intrinsic resistance of B. pseudomallei to many antibiotics complicates treatment. This opportunistic pathogen possesses a wide range of virulence factors, resulting in severe and potentially fatal disease. Virulence factors as targets for drug development offer an alternative approach to combat pathogenic bacteria. Prior to initiating early drug discovery approaches, it is important to demonstrate that disruption of the target gene will prevent the development of disease. This study highlights the fact that KdsD is crucial for virulence of B. pseudomallei in an animal model of infection and provides supportive phenotypic characterization that builds a foundation for future therapeutic development.

## INTRODUCTION

Melioidosis is a potentially fatal disease of both humans and animals caused by the Gram-negative bacterium Burkholderia pseudomallei. While primarily endemic in Southeast Asia and northern Australia, B. pseudomallei is increasingly being isolated from other tropical and subtropical regions and is responsible for a predicted 165,000 melioidosis cases and 89,000 fatalities annually (1). Infection occurs via percutaneous inoculation, ingestion, or inhalation (2) and can present as an acute, chronic, or latent disease (3). Depending on the route of infection, melioidosis can result in diverse symptomatic presentations, including localized skin abrasions, organ abscesses, bacteremia, pneumonia, and septicemia (4, 5). Treatment of infections is complicated by misdiagnosis and the pathogen’s intrinsic resistance to a wide range of antibiotics (6–8). Current treatment typically involves a 2-week intravenous administration of ceftazidime for the acute infection, followed by an eradication phase with co-trimoxazole or co-amoxiclav (9). Treatment duration can last many months (10), increasing the risk of relapse, and, while rare, patient isolates have been recovered that are resistant to ceftazidime and/or co-amoxiclav (11). The absence of a licensed vaccine and a mortality rate that can reach 40% (12) have contributed to the classification of B. pseudomallei as a potential biothreat agent (13). Further improvements in our understanding of the pathogenicity determinates are required to facilitate development of a diverse range of treatments.

Primarily a soil- and surface water-dwelling saprophyte, B. pseudomallei possesses a complex array of virulence factors and secreted effector proteins allowing for colonization and infection of a wide range of animal and plant species (14). These include surface polysaccharides, such as the antigenic lipopolysaccharide (LPS) and capsular polysaccharides (CPS) (15). The LPS (type II O polysaccharide) of B. pseudomallei is a highly immunostimulatory molecule (16) and can provide protection in vaccine efficacy mouse models of melioidosis (17). It is a key virulence determinant primarily due to its important role in the survival of B. pseudomallei within macrophages (18, 19). The B. pseudomallei O antigen has been shown to be a critical component for virulence; an O-antigen mutant displayed increased susceptibility to killing by macrophages because of increased activation of inducible nitric oxide synthase and dysregulation of two defensive Toll-like receptor pathways (19, 20). However, the LPS of *Burkholderia* species has been shown to be structurally and genetically diverse (21, 22). In particular, B. pseudomallei has three distinct O-antigen moieties (serotypes types A, B, and B2) which, while variable, do not correlate with disease severity (23). More widely, the high variability of LPS structures across bacterial genera hinders the identification of conserved genes and sugars for investigation as targets for broad-spectrum therapeutics. However, an exception is the conserved 3-deoxy-d-*manno*-oct-2-ulosonic acid (Kdo), located within the core oligosaccharide (24). The core oligosaccharide is composed of conserved inner components of Kdo and heptose sugars, covalently bound to the lipid A, and a more structurally diverse outer component, bound to the O antigen (24). The minimal LPS structure required for growth in B. pseudomallei is unknown; however, it is largely recognized that lipid A-Kdo (or a Kdo derivative, Ko) is necessary for bacterial growth, although exceptions are known (24). Kdo is also a major constituent of bacterial CPS and exopolysaccharides (EPS), including an EPS isolated from B. pseudomallei, which has been shown to contain repeating units of Kdo and d-galactose (25).

The first committed enzymes in the production of Kdo are d-arabinose 5-phosphate isomerases (APIs), which catalyze the reversible aldol-ketol conversion of d-ribulose 5-phosphate and d-arabinose 5-phosphate. Gram-negative bacteria typically encode at least one API, KdsD, KpsF, or GutQ, with classification being dependent on genomic loci and cellular function. While by definition, the function of these APIs is fixed, the role they play within a bacterium can be variable. For example, KdsD enzymes are associated with production of the LPS (26–28), KpsF enzymes with the production of capsule (29, 30), and GutQ with the regulation of the *gut* operon (31). Multifunctional APIs have been identified, such as KpsF of Neisseria meningitidis, which has a role in both LPS biosynthesis and capsule expression (32). The essentiality of individual API proteins is unclear. For example, while deletion of *kdsD* in E. coli is permissible, further deletion of *gutQ* rendered the cells nonviable, indicating genetic redundancy (33). Pseudomonas aeruginosa PAO1 and Francisella tularensis Schu S4, however, encode only a single API, which is essential only for growth of P. aeruginosa (26, 28). The development of antimicrobials against these well-conserved proteins could allow for inhibition of LPS or EPS assembly, providing a novel antivirulence approach, with the potential to reduce the risk of antimicrobial resistance arising.

B. pseudomallei encodes two APIs: KdsD (BPSL0538) and KpsF (BPSL2770). This study investigated KdsD in B. pseudomallei strain K96243, confirming its enzymatic activity and demonstrating its significant role in virulence. Mutant analysis demonstrated key functions in growth, motility, and survival in RAW 264.7 macrophages and a high level of attenuation in a murine model of melioidosis. Natural suppressor strains of *kdsD* were isolated and genome sequencing revealed mutations in the uncharacterized type IV CPS cluster. This study demonstrates the importance of KdsD for B. pseudomallei virulence and suggests an interconnectivity of API activity on two distinct surface polysaccharides.

## RESULTS

### Bioinformatic and experimental confirmation of BPSL0538 as an arabinose 5-phosphate isomerase.

**FIG 1:**
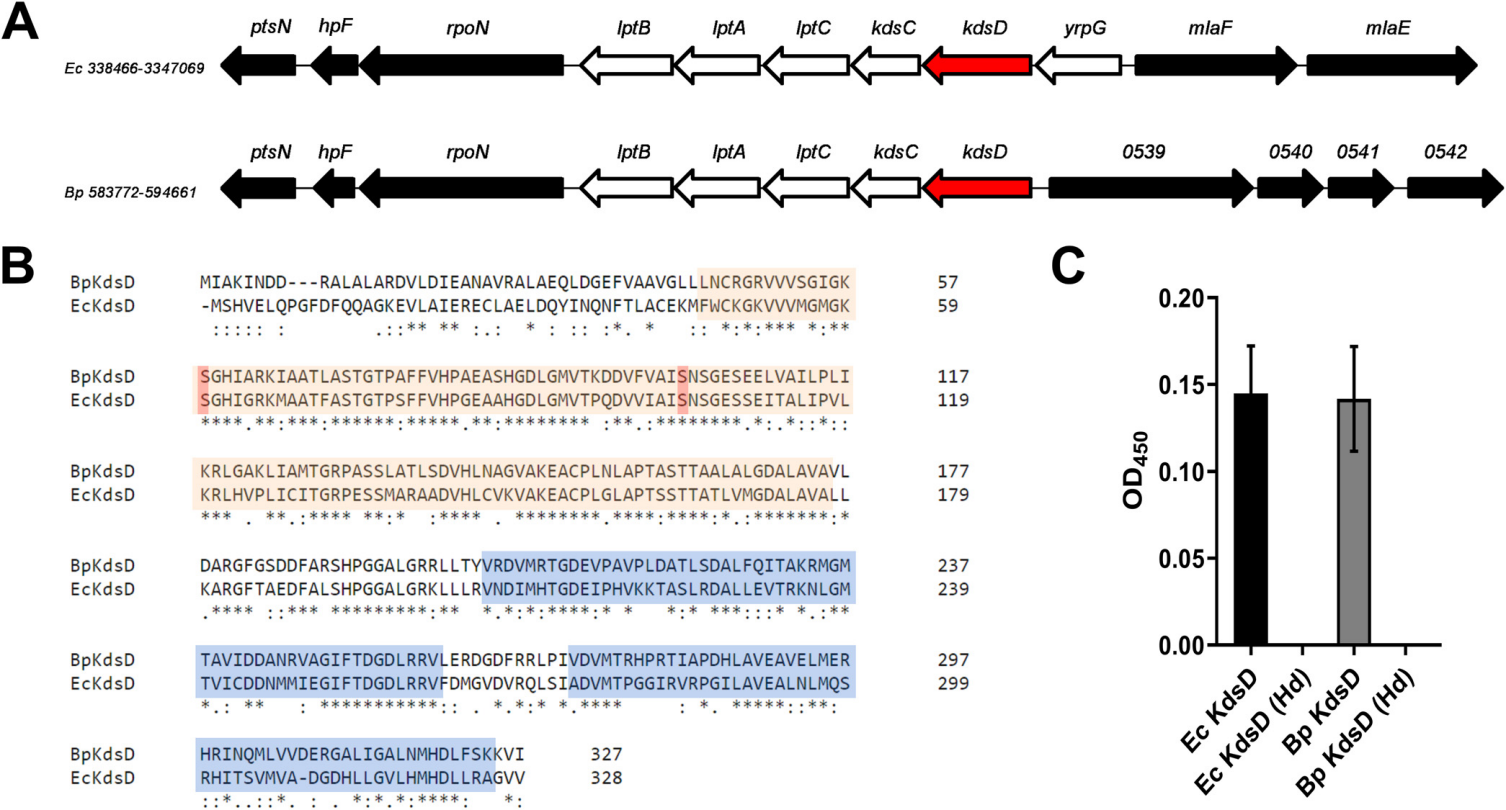
Comparison of sequence/domain homology and activity between KdsD homologues of B. pseudomallei and E. coli. (A) Organization of chromosomal regions encoding KdsD in E. coli (Ec) K-12 substrain MG1655 and B. pseudomallei (Bp) K96243. The E. coli *yrpG*-*lptB* locus and homologous genes in B. pseudomallei are indicated by nonfilled arrows. (B) Amino acid sequences of BpKdsD and EcKdsD were aligned using Clustal to identify conserved residues and domains. Each protein encodes a SIS domain (orange) and two CBS domains (blue). Predicted catalytic cysteine residues are highlighted in red. (C) The API activity of recombinant KdsD was assessed using a discontinuous cysteine-carbazole assay. BpKdsD had API activity similar to that of the control protein EcKdsD. Heat denaturation (Hd) of the proteins abolished the API activity. Experiments were performed in triplicate with four technical replicates per experiment. Error bars represent standard deviations from the means.

BLASTp searches of B. pseudomallei K96243 against API sequences from Escherichia coli identified two APIs: KdsD (BPSL0538), the focus of this study, and KpsF (BPSL2770). *kpsF* is located within a predicted type IV CPS cluster (34, 35), downstream of the well-characterized virulence-associated type I CPS cluster (36–38). *kdsD* is located within the homologous *yrpG-lptB* locus, previously identified in E. coli (39). This locus contains two genes involved in Kdo biosynthesis, *kdsD* and *kdsC*, as well as three of seven genes essential for the transport of LPS to the outer membrane, *lptA*, *lptB*, and *lptC* (Fig. 1A) (40). E. coli K-12 substrain MG1655 also contains *yrpG* within this cluster, encoding a putative Na^+^/Ca^+^ transporter, although we were unable to identify a *yrpG* homologue in B. pseudomallei. KdsD of B. pseudomallei K96243 shares 95% protein sequence coverage with 56.23% homology at the amino acid level to KdsD of E. coli and shares homologous domains, a single catalytic sugar isomerase (SIS) domain (Pfam ID PF01380) and two cystathionine β-synthetase (CBS) domains (Pfam ID PF00571). Two conserved cysteine residues were identified as likely catalytic residues (Fig. 1B). To verify the API activity of B. pseudomallei KdsD, we performed a discontinuous cysteine-carbazole assay. Recombinant KdsD protein from B. pseudomallei was expressed in E. coli BL21(DE3), and purified. *E.coli* KdsD was also used in the assays as a control, along with heat-denatured protein. The relative activities of KdsD from B. pseudomallei and E. coli KdsD were 0.141 and 0.144, respectively. Heat-denatured protein controls did not show any activity (Fig. 1C).

### A KdsD mutant retains an intact LPS and CPS but has increased susceptibility to polymyxin B.

**FIG 2:**
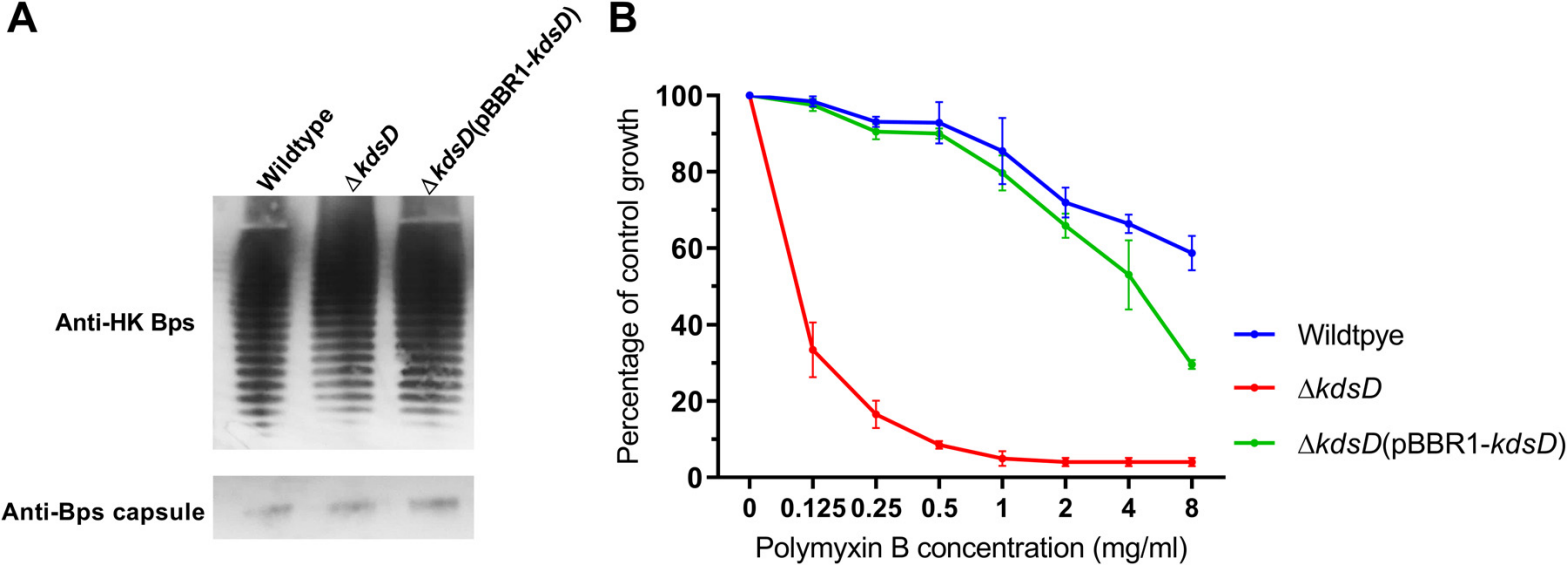
B. pseudomallei Δ*kdsD* displays O-antigen attachment by Western blotting but has increased susceptibility to polymyxin B. (A) Proteinase K-treated whole-cell extracts from the wild type, the Δ*kdsD* mutant, and the Δ*kdsD*(pBBR1-*kdsD*) strain were analyzed by Western blotting using either anti-B. pseudomallei (Bps) LPS antibody or an anti-B. pseudomallei CPS antibody. Extracts display typical LPS laddering profiles, indicating assembly of O antigen onto the lipid A and the presence of capsular polysaccharide. (B) Growth of cultures containing increasing concentrations of polymyxin B was measured by OD, and the percentage of growth compared to a no-antimicrobial control was calculated. The Δ*kdsD* mutant was >16 times more susceptible to polymyxin B than the wild-type strain. Data are means for three biological replicates.

Given the locus of *kdsD* within a Kdo-biosynthetic cluster, we hypothesized that its deletion would result in incomplete formation of the LPS core and O antigen. To investigate this, we generated an unmarked deletion mutant, the Δ*kdsD* mutant. A complementing strain was also generated by the introduction of pBBR1MCS1*-kdsD* into the Δ*kdsD* mutant [Δ*kdsD*(pBBR1*-kdsD*)] by conjugation. To investigate the presence of O antigen, Western blotting of proteinase K-digested whole-cell lysates was performed using anti-B. pseudomallei LPS monoclonal antibodies (Fig. 2A). There were no differences observed between the wild-type and Δ*kdsD* samples, with both strains producing the characteristic LPS ladder pattern, indicative of the presence of polysaccharide. Qualitative analysis using antibodies targeting B. pseudomallei CPS revealed that all study strains retained CPS (Fig. 2A). To determine potential defects in the protective properties of LPS, we assessed the susceptibility of the *kdsD* deletion mutant to the cationic peptide polymyxin B (Fig. 2B). Deletion of *kdsD* resulted in increased susceptibility to polymyxin B (*P < *0.0001) at all concentrations tested, with visible growth (optical density [OD]) of the wild type and the Δ*kdsD*(pBBR1*-kdsD*) strain at concentrations up to 8,000 μg/mL, while there was limited or no growth of the Δ*kdsD* strain at concentrations of >500 μg/mL. Restoration of susceptibility to polymyxin B through complementation was only partial at higher concentrations.

### The Δ*kdsD* mutant has a temperature-dependent growth and motility defect and has attenuated survival in RAW 264.7 macrophages.

**FIG 3:**
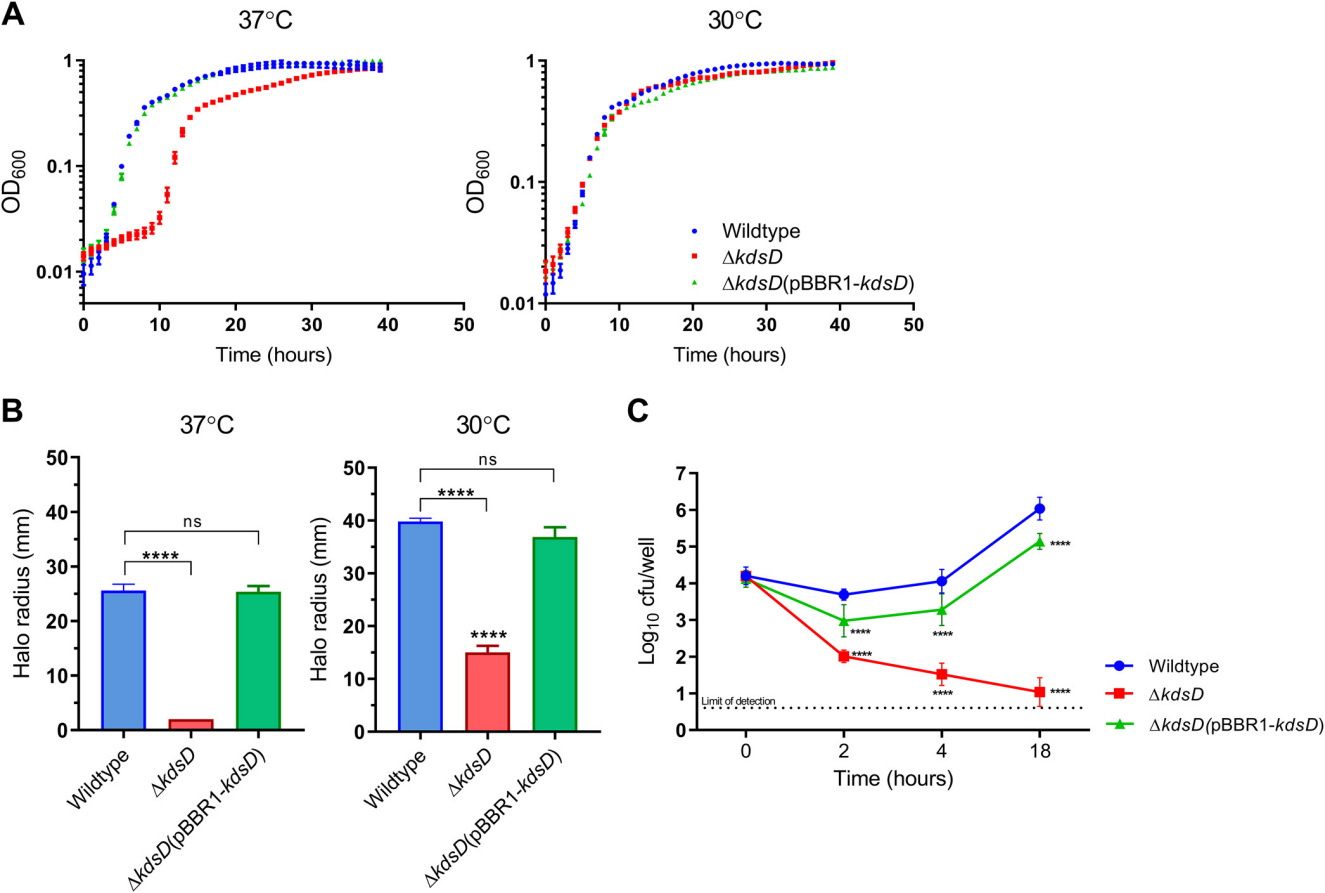
The Δ*kdsD* mutant has temperature-dependent growth and motility defects and is attenuated in murine-derived macrophages. (A) Growth in LB medium was assessed at 37°C and 30°C in a 96-well plate, with shaking. Growth was monitored by measurement of OD_600_ at 30-min time points. Growth at 37°C resulted in an extended lag phase compared to growth at 30°C. (B) The swimming motility of the B. pseudomallei wild type, the Δ*kdsD* mutant, and the Δ*kdsD*(pBBR1-*kdsD*) strain was assessed by growth on semisolid agar. The halo radius was measured after 24 h (37°C) or 36 h (30°C). The Δ*kdsD* mutant displayed significant reduction in motility at both 37°C and 30°C. (C) RAW 264.7 murine macrophages were infected with B. pseudomallei strains at an MOI of 1. Bacteria were enumerated by CFU counts at 0, 2, 4, and 18 h postinfection. All strains had similar concentrations of intracellular bacteria 1 h postinfection (*T*_0_). The Δ*kdsD* mutant had significantly reduced survival at all subsequent time points. Data are from four independent experiments with two technical replicates per experiment; results were combined and log transformed. One-way ANOVA comparing the wild type to both the mutant and complemented strain was performed for all experiments (****, *P* < 0.0001; ns, not significant). Comparison of the mutant and complemented strain showed partial restoration of wild-type phenotypes in all assays (*P* < 0.0001). Error bars indicate standard deviations from the means.

Given the increased susceptibility of the Δ*kdsD* mutant to polymyxin B, despite having an apparently complete oligomeric O-antigen attachment visualized by the Western blot, we aimed to investigate the impact of mutation on a wider array of biological processes. Growth studies were performed using nutrient-rich medium at 30°C and 37°C. The Δ*kdsD* mutant demonstrated an extended lag phase of growth, a defect observed only at 37°C, with growth at 30°C being equivalent to that of the wild-type strain (Fig. 3A). Complementation restored a wild-type pattern of growth. Assessment of swimming motility also demonstrated significant temperature-dependent defects in the Δ*kdsD* mutant (Fig. 3B). Culturing of the Δ*kdsD* mutant at 37°C revealed a nonmotile phenotype (wild type, 25.6 ± 1.1 mm; Δ*kdsD* mutant, 2.0 ± 0.0 mm [*P < *0.0001]; Δ*kdsD*(pBBR1*-kdsD*) strain, 25.4 ± 1.0 mm [*P = *0.8218]), whereas culturing at 30°C resulted in a motile phenotype, although significantly reduced from that of the wild type (wild type, 39.8 ± 0.6 mm; Δ*kdsD* mutant, 15.0 ± 1.3 mm [*P < *0.0001], Δ*kdsD*(pBBR1*-kdsD*) strain, 36.9 ± 1.8 mm [*P < *0.0001]).

The effect of a *kdsD* deletion on infection and survival in RAW 264.7 macrophages was assessed using a kanamycin protection assay (Fig. 3C). Cells were lysed at 0, 2, 4, and 18 h postinfection, and intracellular bacteria were enumerated by CFU counts. At *T*_0_, there was no significant difference between the counts of internalized bacteria for wild-type- or Δ*kdsD* mutant-infected cells (*P* = 0.997), indicating comparable uptake for both strains. Following entry, the Δ*kdsD* mutant exhibited a reduced ability to survive and replicate at 2, 4, and 18 h (*P < *0.0001). At 18 h postinfection, a mean 5-log decrease in intracellular bacteria between wild-type- and Δ*kdsD* mutant-infected cells was observed. Complementation of *kdsD* resulted in restoration of bacterial survival, with the complementing strain showing intracellular replication at the 18-h time point.

### Suppressor mutations in the type IV CPS cluster partially restore motility defects.

**FIG 4:**
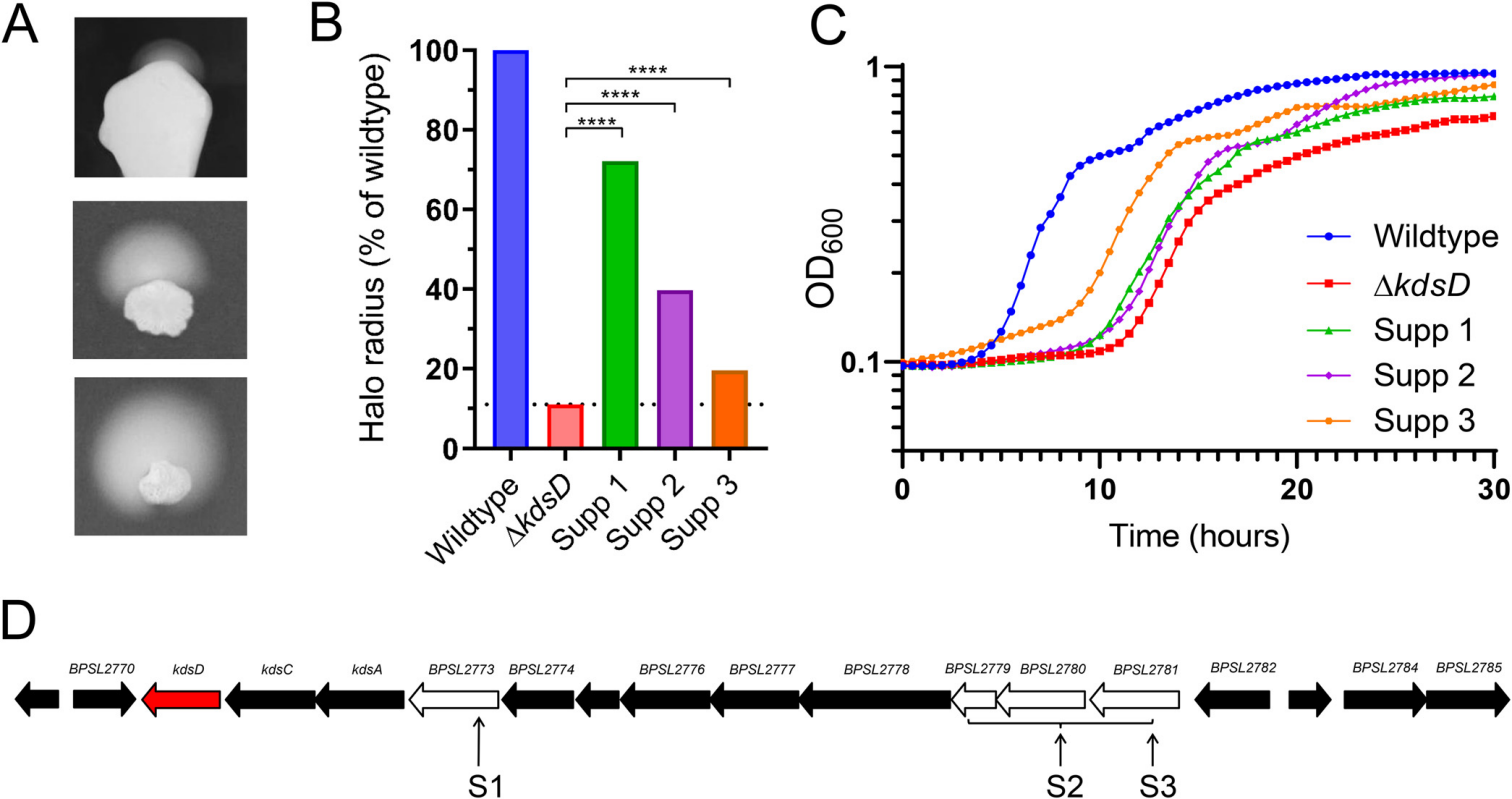
Suppressor mutations were identified in the CPS type IV cluster. (A) Representative images of suppressor mutants with a reverted motility phenotype at 37°C. (B) Isolation and characterization of three suppressor mutants revealed various degrees of reversion to the wild-type phenotype. Data are percentages of wild-type motility from three repeat experiments. The three suppressor strains had a significant increase in motility compared to the Δ*kdsD* mutant (one-way ANOVA; ****, *P* < 0.0001). Only partial restoration of the wild-type motility phenotype was seen, with all suppressor strains having significantly less motility than the wild-type strain (one-way ANOVA, ****, *P* < 0.0001). (C) Growth in LB medium was assessed at 37°C in a 96-well format. Suppressor strains had only partial recovery of the growth defect. (D) Loci of SNPs or deletions in Δ*kdsD* suppressor mutants (S1, S2, and S3) are located within the type IV CPS cluster.

During the evaluation of *in vitro* phenotypes, we observed that extended incubation (4 to 5 days) of motility plates inoculated with the Δ*kdsD* mutant resulted in the appearance of small extrusions in which motility had been restored (Fig. 4A). To investigate the nature of these revertants, bacteria were isolated and subcultured, and motility assays were performed. The three strains isolated exhibited various degrees of reversion relative to the wild-type phenotype: suppressor 1 (S1), 70.5% reversion; S2, 38.8% reversion; and S3, 21.3% reversion (Fig. 4B). The growth defect observed at 37°C was partially restored in the three suppressor strains, with entry into exponential phase decreasing from 11 h for the Δ*kdsD* mutant to 10, 10, and 8 h for suppressors 1, 2, and 3, respectively (Fig. 4C). Interestingly, the growth restoration was inversely restored relative to motility restoration, i.e., suppressor strains with the highest growth restoration at 37°C exhibited the lowest motility restoration.

All three strains revealed suppressor mutations in the uncharacterized type IV CPS cluster encoded by BPSL2769 to BPSL2785 (Table 1; Fig. 4D). Importantly, this gene cluster encodes the second API, KpsF (BPSL2770). Attempts to delete *kpsF* from the genome of the Δ*kdsD* mutant failed despite repeated efforts. Protein sequences of mutated genes were submitted for pairwise comparison using the EMBOSS Needle algorithm V6.6.0 (41), revealing 39.5% identity and 49% similarity of BPSL2779 (Q63R94) to E. coli Wzb (P0AAB2), 32.0% identity and 50.5% similarity of BPSL2780 (Q63R63) to Wza (P0A930), and 26.1% identity and 39.3% similarity of BPSL2781 (Q63R92) to WcaJ (P71241). The putative glycosyltransferase (Pfam domains PF00534 and PF13439) BPSL2773 (Q63RA0) had 23.9% identity and 37.9% similarity to gene encoding an uncharacterized 41.2-kDa protein located directly downstream of *wza*, *wzb*, and *wxc* in the CPS region of Klebsiella pneumoniae (Q48453).

**TABLE 1:** Summary of SNPs and deletions in *kdsD* motile suppressor mutants

Strain	Location	SNP or indel	Protein mutation	Gene and protein function
Suppressor 1	BX571965: 3306074	SNP (T > G)	NA^*a*^	Intergenic, noncoding
	BX571965: 3310736	Del (CA>C)	Frame shift	BPSL2773, glycosyl transferase family 1
Suppressor 2	BX571965: 3318000–3320455	Del	Deletion	BPSL2779, protein tyrosine phosphatase; BPSL2780, capsular polysaccharide transport protein; BPSL2781, undecaprenyl-phosphate galactose phosphotransferase
Suppressor 3	BX571965: 1471427	SNP (A > C)	Leu46Arg	BPSL1269, IclR family transcriptional regulator
	BX571965: 3319687	SNP (G > A)	Thr323Met	BPSL2781, transmembrane sugar transferase

a NA, not applicable.

### A *kdsD* mutant is attenuated in a BALB/c mouse model of melioidosis.

**FIG 5:**
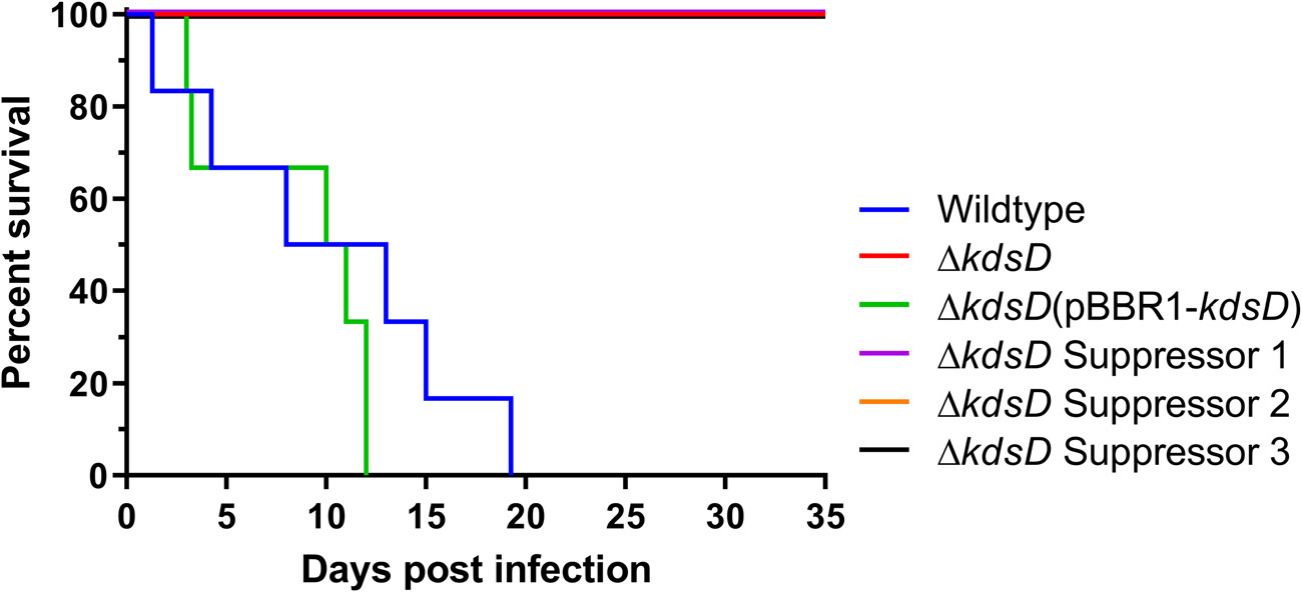
The Δ*kdsD* mutant is highly attenuated in a mouse model of melioidosis, and natural suppressor mutations do not result in reversion of attenuation. BALB/c mice (*n* = 6) were infected via the intraperitoneal route with approximately 1 × 10^5^ CFU (approximately 130 median lethal doses [MLD]) or a higher challenge dose of 1 × 10^6^ CFU [actual received doses: wild type, 1.0 × 10^5^ CFU; Δ*kdsD* mutant, 1.2 × 10^6^ CFU; Δ*kdsD*(pBBR1-*kdsD*) strain, 8.0 × 10^4^ CFU; Δ*kdsD* suppressor 1, 8.0 × 10^5^ CFU; Δ*kdsD* suppressor 2, 1.2 × 10^6^ CFU; Δ*kdsD* suppressor 3, 1.0 × 10^6^ CFU]. Deletion of *kdsD* resulted in significant attenuation, with all mice surviving to the end of the study (Mantel-Cox test, *P* < 0.0001). Suppressor mutations did not restore virulence.

To determine the role of KdsD in virulence, the Δ*kdsD* mutant and associated suppressor strains were assessed in an intraperitoneal mouse model of melioidosis (Fig. 5). At 35 days postinfection, surviving mice were culled, spleens were aseptically removed, and the bacterial load in the spleen was enumerated by colony counts. All mice challenged with the wild-type strain succumbed to infection by day 19, with a median survival of 10.5 days. The Δ*kdsD* mutant was significantly attenuated (Mantel-Cox test, *P < *0.0001), with all mice surviving to the end of the study, including those challenged with a 10-fold-higher dose of 1 × 10^6^ CFU. There were no culturable bacteria in the spleens of the surviving mice, indicating clearance of the Δ*kdsD* mutant by the host. Virulence was restored through complementation of *kdsD*, with all mice succumbing by day 12, with a median survival also of 10.5 days. Notably, there was no mortality or morbidity in any mice challenged with the suppressor strains, again at the higher challenge dose, and all mice had cleared the infection from the spleen.

## DISCUSSION

B. pseudomallei is a saprophytic bacterium capable of causing debilitating and fatal infection. Its ability to resist a range of antibiotic treatments and potential for relapse or establishment of latent infection (3) mean that alternative approaches are required to rapidly treat melioidosis. One emerging approach is to target bacterial virulence. This strategy could offer substantial benefits and reduced risks relative to treatments that target essential proteins, such as reducing selection pressure and associated resistance, protecting the gut microbiome, and, in the case of repurposing of approved drugs, reducing the need for costly and lengthy discovery programs (42–45). One family of proteins that have previously been shown to be important virulence determinants in other biothreat agents, including F. tularensis (26, 46) and Yersinia pestis (47), are arabinose-5-phosphate isomerases. Some efforts have been made to generate inhibitors against KdsD or APIs (48–51) previously, and this study now demonstrates a crucial role for the API KdsD in B. pseudomallei virulence, validating it as a potential target for the future development of novel broad-spectrum antimicrobial compounds for the treatment of melioidosis.

Consistent with the shared protein sequence homology between B. pseudomallei BPSL0538 and E. coli KdsD (including domain architecture and predicted catalytic sites), this study has established that BPSL0538 also exhibits similar API activity, confirming its role as KdsD. Deletion of *kdsD* resulted in temperature-dependent *in vitro* phenotypes, including increased lag phase growth and motility defects. More pronounced phenotypes were observed at the higher temperature more typically associated with the host environment. Temperature-related phenotypes have been described previously in E. coli LPS core mutants (52, 53), but the exact reason for this temperature-dependent phenotype is unknown. Deletion of *kdsD* significantly attenuated B. pseudomallei survival within RAW 264.7 macrophages and increased susceptibility to the membrane-destabilizing cationic peptide polymyxin B. These phenotypes are consistent with analyses of LPS-defective mutants of B. pseudomallei reported previously (19, 20, 54, 55). Visualization of proteinase K-treated whole-cell extracts by Western blotting revealed that deletion of B. pseudomallei *kdsD* was not sufficient to prevent attachment of the O antigen. We hypothesized that the absence of Kdo linking lipid A to the inner core would cause the loss of O antigen similar to that observed following disruption to the core oligosaccharide in Burkholderia cenocepacia (56) and observed for a *kdsD* deletion strain generated in F. tularensis (26). However, F. tularensis encodes only a single API (26), in contrast to B. pseudomallei, which possesses KpsF, with 48.1% identity to KdsD. Therefore, KpsF may be able to provide sufficient API activity for O-antigen attachment. In-depth analysis of the LPS structure could help to elucidate why these phenotypes differ despite apparent full LPS assembly. Our attempts to make a KdsD KpsF double deletion mutant were unsuccessful; this suggests that synthesis of Kdo may be essential in B. pseudomallei, although this has not been confirmed. Similarly, while both *kdsD* and *gutQ* could be individually deleted in E. coli, a double API mutant of E. coli was nonviable (33).

The *in vitro* phenotypes observed following *kdsD* deletion are likely contributors to the significant attenuation observed *in vivo*. The effects observed were pleiotropic, and it is possible that reduced growth of the mutant at 37°C could play a significant role. The clearance of the *kdsD* mutant from mice demonstrates a clear role of KdsD in the pathogenesis of B. pseudomallei. Mice challenged with the Δ*kdsD* mutant did not display typical clinical signs that often occur even with attenuated mutants. This suggests rapid clearance by the innate immune system rather than the slower activation of B cells and the production of antigen-dependent and antigen-specific antibodies; therefore, the efficacy of the Δ*kdsD* mutant for use as a live attenuated vaccine may be limited. The isolation of suppressor strains with *in vitro* phenotypic reversion raised the concern that should this pathway be targeted by therapeutic intervention, emergence of resistance may be rapid. However, the suppressor strains retained full attenuation within the mouse model of infection, suggesting that this may not represent a route for drug resistance.

Investigations into the Δ*kdsD* suppressor strains revealed mutations in the predicted type IV CPS cluster (34, 35), a region that also includes the gene for the second putative B. pseudomallei API, *kpsF*. Within this cluster are a number of genes (*wza*, *wzb*, and *wzc*) that are conserved across a range of genera, including, but not restricted to, E. coli, Vibrio vulnificus, and Klebsiella species (57–59). In this system, newly synthesized CPS is exported through Wza, an outer membrane translocon, aided by Wzc, a tyrosine autokinase, and Wzb, a tyrosine phosphatase, located on the inner membrane (60). Wza exhibits limited specificity for the translocated sugar and can therefore export CPS with significant structural diversity (61), suggesting an explanation for conservation of this system across bacteria producing diverse polysaccharides. Other mutations arose in less conserved CPS genes, including a homologue of WcaJ that is known to initiate the synthesis of CPS and the various glycosyltransferases (potentially including BPSL2773) that process CPS to maturation (62). Two other single nucleotide polymorphism (SNP) mutations were identified in these suppressor strains, one of which was in an intergenic region, but it is not clear if these contribute to phenotype suppression or are a consequence of prolonged growth. B. pseudomallei is known to accumulate mutations in laboratory stocks, with changes to BPSL1269 also having been reported (63).

The paucity of research into the type IV CPS cluster in B. pseudomallei makes it challenging to derive conclusive explanations for some of the observations reported here. The absence of KdsD could simply generate a cellular deficiency in Kdo that is partially restored by disruption of a competing capsule pathway. This is consistent with our observation that phenotypes become more pronounced at higher temperatures, where a more rapidly dividing cell is likely to create a greater demand for Kdo. Alternatively, it is possible that KdsD is also required for synthesis of type IV CPS and that the subsequent accumulation of CPS intermediates negatively impacts the cell. To our knowledge, there is no evidence of a role in virulence for the type IV CPS cluster and it has not been identified in whole-genome transposon infection screens (64). In addition, it is not encoded by the closely related, host-adapted species Burkholderia mallei. These observations highlight the need for further in-depth functional studies into the type IV CPS system and its role in the biology of B. pseudomallei.

In conclusion, this is the first study into KdsD of B. pseudomallei; we have demonstrated its importance for B. pseudomallei virulence, providing a new candidate for future antimicrobial development. This study highlights an intricate relationship between two polysaccharides produced by B. pseudomallei, characterization of which is beyond the scope of this study. Further research is therefore required to understand the complex polysaccharides produced by *Burkholderia*, including the neglected type III and IV CPS and the importance of paralogous genes within different exopolysaccharide clusters, which may have significant synergistic effects.

## MATERIALS AND METHODS

### Bacterial strains, media, and growth conditions.

All strains can be found in Table 2; primers used in this study can be found in Table S1 in the supplemental material. Studies involving the use of B. pseudomallei were performed in advisory committee on dangerous pathogens (ACDP)/advisory committee on genetic modification (ACGM) level 3 laboratories at the Defense Science and Technology Laboratory (DSTL). Cultures of B. pseudomallei were prepared by suspending a loopful of B. pseudomallei, grown on lysogeny agar (LA), in 10 mL of phosphate-buffered saline (PBS). The optical density (OD) at 600 nm was adjusted to 1.0, and 1 mL was used to inoculate 50 mL of lysogeny broth (LB). Cultures were incubated at 37°C with shaking overnight. E. coli cultures were prepared by inoculation of LB with an isolated colony or from a glycerol stock followed by incubation at 37°C with shaking overnight. Where appropriate, medium was supplemented with kanamycin (E. coli, 50 μg/mL; B. pseudomallei, 800 μg/mL), chloramphenicol (50 μg/mL), or ampicillin (100 μg/mL). Unless otherwise stated, media and reagents were purchased from Sigma-Aldrich or Thermo Fisher Scientific.

**TABLE 2:** Strains used in this study

Strain	Description^*a*^	Source or reference
E. coli		
BL21(DE3)	BL21 containing λDE3 lysogen	New England Biolabs
BL21(DE3)::pBAD/HisB-BpKdsD	BL21(DE3) containing pBAD/HisB-BpKdsD for recombinant expression of B. pseudomallei KdsD	This study
BL21(DE3)::pBAD/HisB-EcKdsD	BL21(DE3) containing pBAD/HisB-EcKdsD for recombinant expression of E. coli KdsD	This study
S17-1 (λ*pir*)	S17-1 containing *pir* encoding λ prophage	80
B. pseudomallei		
K96243	Clinical isolate, Thailand, 1996	34
K96243Δ*kdsD*	Unmarked deletion in BPSL0538	This study
K96243Δ*kdsD*(pBBR1*-kdsD*)	Unmarked deletion in BPSL0538::pBBR1MCS-1*-kdsD* (Chl^r^)	This study
K96243Δ*kdsD-*S1	Unmarked deletion in BPSL0538; spontaneous mutation in BPSL2773	This study
K96243Δ*kdsD-*S2	Unmarked deletion in BPSL0538; spontaneous mutations in BPSL2779, BPSL2780, and BPSL2781	This study
K96243Δ*kdsD-*S3	Unmarked deletion in BPSL0538; spontaneous mutation in BPSL1269 and BPSL2781	This study

a BPSL numbers are locus tags for the B. pseudomallei K96243 genome.

### Expression and purification of recombinant KdsD from B. pseudomallei and E. coli.

KdsD encoding regions were PCR amplified using B. pseudomallei strain K96243 or E. coli strain MG1655 (65) genomic DNA as a template. PCR products were inserted between the restriction sites HindIII and BglII on the expression vector pBAD/HisB by Gibson assembly. The two constructs, pBAD/HisB-BpKdsD and pBAD/HisB-EcKdsD, were sequenced and used in the transformation of E. coli BL21(DE3) (New England Biolabs). Recombinant API expression strains were grown in LB supplemented with ampicillin to an OD at 600 nm of 0.6 to 0.8. Cultures were induced with l-arabinose at a final concentration of 0.5% (wt/vol). After 4 h, the bacteria were harvested by centrifugation (6,000 × *g*, 20 min, 4°C). Pellets were resuspended in buffer A (25 mM Tris HCl [pH 8], 500 mM NaCl) and lysed by sonication (four 30-s bursts). Cell debris was removed by centrifugation (25,000 × *g*, 20 min, 4°C). His-tagged proteins were captured by affinity chromatography using HisTrap HP columns (GE Healthcare). The column was equilibrated with buffer A before addition of the clarified cell lysate. Non-specifically bound proteins were removed by repeat column washes in buffer A containing 100 mM imidazole. Recombinant API proteins were eluted in buffer A containing 250 mM imidazole. Protein fractions were dialyzed against buffer A at 4°C. SDS-PAGE analysis confirmed that the purity of the protein was >90%. Western blotting using antipolyhistidine antibodies was performed to confirm the identity of purified proteins. Recombinant protein was stored at −80°C until use.

### Determination of API activity.

API activity was determined using a discontinuous cysteine-carbazole colorimetric assay as described previously (27, 66). Briefly, 25 μL of recombinant enzyme at 3 nM in 100 mM Tris HCl (pH 8.5) was incubated for 3 min at 37°C in a thermocycler (Bio-Rad). Twenty-five microliters of 20 mM arabinose 5-phosphate in 100 mM Tris HCl (pH 8.5) was added. The reaction was stopped after 3 min by the addition of 50 μL of 25 N H_2_SO_4_. Ninety microliters was transferred to a flat-bottomed assay plate containing 250 μL of 25 N H_2_SO_4_–cysteine carbazole solution (230 μL 25 N H_2_SO_4_ plus 10 μL aqueous cysteine [1.5% {wt/vol}] and 10 μL of a 0.12% [wt/vol] ethanolic carbazole solution [carbazole was dissolved in 100% ethanol]). The solution was mixed thoroughly and left for 1 h at room temperature. Absorbance changes were measured at 540 nM. Experiments were performed in triplicate with four technical replicates per experiment.

### Generation of an unmarked *kdsD* deletion mutant and complemented strain.

A *kdsD* unmarked deletion mutant was generated by allelic exchange using the suicide vector pMo130 (67). The upstream flanking region (FR1) and downstream flanking region (FR2) were PCR amplified using B. pseudomallei K96243 genomic DNA with KdsD FR1 and FR2 primer sets (Table S1). PCR products were cloned in a three-way ligation between the HindIII and XbaI sites of pMo130, using a shared BamHI site between flanking regions. The final construct, pMo130*-kdsD*, was electroporated into E. coli S17-1 (λ*pir*). Conjugal transfer was performed as described previously (67) with the following modifications; single crossovers were selected on kanamycin (800 μg/mL) and polymyxin B (15 μg/mL), and double-crossover mutants were selected for by using 20% (wt/vol) sucrose. Deletion mutants were confirmed by PCR using flanking and gene-specific primers followed by sequencing. For in *trans* complementation of the Δ*kdsD* mutant, the *kdsD* open reading frame was PCR amplified using primers KdsD_Comp_For and _Rev and cloned between the HindIII and BamHI sites of pBBR1-MCS1 (Chl^r^) (68) to generate the plasmid pBBR1MCS1*-kdsD*. The plasmid was conjugated into the B. pseudomallei Δ*kdsD* mutant as outlined above [Δ*kdsD*(pBBR1*-kdsD*)]. Antibiotic and inducer were not essential for maintenance of the plasmid (data not shown); therefore, to allow consistent growth conditions between strains, these were omitted from culture media for all experiments. A further construct for the deletion of *kpsF* was created using the approach detailed above in an attempt to generate a *kdsD kpsF* double-deletion strain.

### Growth assays.

Growth curves were performed using LB medium in 96-well plates using a Multiskan FC microplate photometer (Thermo Fisher Scientific) using the continuous background shaking setting. Overnight cultures were adjusted to an OD of approximately 0.2. Cultures were diluted 10-fold in LB, and 20 μL was inoculated into 180 μL of LB in triplicate wells of a 96-well microtiter plate. Plates were incubated at either 30°C or 37°C with continuous shaking, and OD measurements were recorded every hour for 36 h. Blank control wells were included, and the mean OD for blank wells was subtracted from each reading. Experiments were repeated three times, with four replicates per experiment, and the data were combined.

### Motility assays.

Swimming motility assays were performed using LA plates containing 0.3% agar. Ten milliliters of LB was inoculated with each strain and incubated at 37°C with shaking at 180 rpm overnight. The OD of each strain was adjusted to 0.2 (approximately 1 × 10^8^ CFU/mL), and 2 μL was inoculated into the center of the 0.3% agar plates. Plates were incubated at either 30°C or 37°C for 16 or 24 h, respectively. Motility was assessed by measuring the halo radius. Three biological replicates were performed, with three technical replicates per experiment.

### Cellular uptake and intracellular survival assays.

Bacterial uptake and survival were quantified using a modified kanamycin protection assay as described previously (69). RAW 264.7 macrophages were routinely cultured in Dulbecco’s modified Eagle medium supplemented with 2 mM l-glutamine and 10% fetal bovine serum (FBS) and maintained at 37°C and 5% CO_2_ in a 95% humidity incubator. Twenty-four-well flat-bottomed plates were seeded with 5 × 10^5^ cells and incubated for 18 h to allow cells to adhere. One hour prior to infection, the culture medium was removed and replaced with 1 mL of Leibovitz's L-15 medium and GlutaMAX (Thermo Fisher Scientific) containing 10% FBS and incubated at 37°C. Overnight bacterial cultures were adjusted to an OD_590_ of 0.2 in L-15 medium and serially diluted. L-15 medium was removed from the macrophages and replaced with 1 mL of B. pseudomallei at a multiplicity of infection (MOI) of 1. Cells were infected at 37°C for 1 h. Macrophages were washed once in prewarmed PBS and incubated in 1 mL L-15 medium supplemented with kanamycin (800 μg/mL) to kill remaining extracellular bacteria (time zero). At 2, 4, and 18 h, culture medium was removed, and the cells were lysed in 1 mL of distilled water (dH_2_O) and enumerated by CFU counts on LA. Four biological replicates were performed, with two technical replicates per experiment.

### LPS profiles of whole-cell extracts.

Approximately 1 mg of B. pseudomallei harvested from an LA plate following 48 h of growth was suspended in 500 μL of solubilization buffer (62.5 mM Tris-HCl [pH 6.8], 10% glycerol, 3% SDS, 4% 2-mercaptoethanol) and heated at 98°C for 15 min. Unlysed cells and debris were separated by centrifugation at 5,000 × *g* for 2 min. The supernatant was transferred to a clean tube, and DNA and RNA were removed by the addition of 20 μg/mL of DNase and 20 μg/mL of RNase followed by incubation at 37°C for 3 h. Protein was removed through the addition of 50 μg/mL proteinase K and incubation at 60°C for 3 h. To visualize the LPS profile of B. pseudomallei strains, Western blot analysis was performed using an anti-B. pseudomallei LPS antibody. Western blotting was also performed to demonstrate the presence of capsular polysaccharide using an in-house anti-B. pseudomallei capsule monoclonal antibody (70).

### Susceptibility to polymyxin B.

Overnight cultures were grown in Mueller-Hinton broth (MHB) before adjustment to an OD_600_ of 0.2 in fresh MHB. Approximately 1 × 10^5^ CFU was added to 5 mL of MHB containing polymyxin B (2-fold dilution series from 125 μg/mL to 8,000 μg/mL). Cultures were incubated for 24 h at 37°C with shaking at 180 rpm, and the OD was recorded. Experiments were performed in triplicate, and data are presented as percent change relative to an antibiotic-free control to account for a growth defect exhibited by the Δ*kdsD* mutant at 37°C.

### Whole-genome sequencing.

Genomic DNA was extracted using a Gentra Purgene DNA extraction kit (Qiagen) following the manufacturer’s instructions. Samples were prepared for sequencing using the Illumina TruSeq DNA PCR-free library preparation kit and sequenced on an Illumina MiSeq instrument. Adapter sequences and low-quality bases (<*Q*22) were removed using cutadapt (71) version 2.5. Reads before and after trimming were checked for quality using FastQC (72), and for common contaminants using FastQ Screen (73). MultiQC was used to collate and visualize the results (74). A subset of reads was also checked with BLAST (75) against the NCBI nucleotide database for other contaminants. Results were visualized using Krona (76). The trimmed reads were aligned against the GenBank assembly (77) accession GCA_000011545.1 using Burrows-Wheeler Aligner (BWA) (77) (>100× coverage depth) and processed, and SNPs were identified according to Genome Analysis Toolkit (GATK) best practices (78). Larger indels were identified using DELLY (79).

### Murine infection model.

Experiments involving animals were carried out according to the requirements of the UK Animal (Scientific Procedures) Act 1986. This project license was approved following an ethical review by DSTL’s Animal Welfare and Ethical Review Body.

Six- to 8-week-old female BALB/c mice were obtained from Charles River (Margate, UK). The mice were randomized, housed in groups of 6, and allowed to acclimate for 7 days. B. pseudomallei strains used for the challenge were grown as outlined above. The OD of the overnight cultures was adjusted to 0.2 (approximately 1 × 10^8^ CFU/mL) before serial dilution to the appropriate concentration for challenge in PBS. Mice were infected via the intraperitoneal route and observed for a 35-day period following strict predetermined humane end points. At the end of the study, surviving mice were culled, and the spleens were aseptically removed. Each spleen was weighed and homogenized in 1 mL PBS, and bacteria were enumerated by CFU counts on LA. Bacterial clearance from the spleens was confirmed by adding the remaining tissue homogenates to 10 mL LB and incubating for 72 h at 37°C. These were streaked on LA to look for the presence of B. pseudomallei colonies.

### Statistical analysis.

Data analysis and production of figures were performed using GraphPad Prism version 8.0.1. A log-rank (Mantel-Cox) test was used for analysis of *in vivo* data. One-way analysis of variance (ANOVA) was performed for motility and macrophage infection studies.
