# Supplementary material for: The Arabinose 5-Phosphate Isomerase KdsD Is Required for Virulence in Burkholderia pseudomallei
Source: J Bacteriol. 2023 Jul 17;205(8):e00034-23. doi: 10.1128/jb.00034-23 (PMC10448790; doi:10.1128/jb.00034-23)
Supplement: Supplemental file 1 — Table S1. Download jb.00034-23-s0001.docx, DOCX file, 0.01 MB [file jb.00034-23-s0001.docx]

**Supplementary material**

*Table S1: Primers used in this study*

| KdsD_For | AGACATATGATAGCGAAAATCAATGATGATCGG | KdsD overexpression | NdeI |
| --- | --- | --- | --- |
| KdsD_Rev | ACAGGATCCTCAAATCACCTTCTTCGAGAACAG | KdsD overexpression | BamHI |
| KdsD_Comp_For | TACAAGCTTATGATAGCGAAAATCAATGATGATCGG | PCR of KdsD encoding region | HindIII |
| KdsD_Comp_Rev | TATGGATCCTCAAATCACCTTCTTCGAGAAC | PCR of KdsD encoding region | BamHI |
| KdsD_FR1_For | AAGCTTAGCAGCACGCCGAAGATGTTG | PCR amplification of FR1 for KdsD deletion mutant | HindIII |
| KdsD_FR1_Rev | GGATCCGAGCGCCCGATCATCATTGAT | PCR amplification of FR1 for KdsD deletion mutant | BamHI |
| KdsD_FR2_For | GGATCCAACATGCACGATCTGTTCTCG | PCR amplification of FR2 for KdsD deletion mutant | BamHI |
| KdsD_FR2_Rev | TCTAGATCGCCTGCAGCAGCCAGTACG | PCR amplification of FR2 for KdsD deletion mutant | XbaI |
| KpsF_FR1_For | TATAAGCTTGAAGGACGGCATCGCCGTTGCGCT | PCR amplification of FR1 for KpsF deletion mutant | HindIII |
| KpsF _FR1_Rev | TATCTCGAGTCGCACGCACGCTGCATGAAGCC | PCR amplification of FR1 for KpsF deletion mutant | XhoI |
| KpsF _FR2_For | TATCTCGAGAAGAAATAAGGAGGCGGCACGCGG | PCR amplification of FR2 for KpsF deletion mutant | BamHI |
| KpsF _FR2_Rev | TATGGATCCGCTACGTGCTCACGCCGCGCATCTTCG | PCR amplification of FR2 for KpsF deletion mutant | XhoI |
| KdsD_GS_For | TGTTCCAGATCACCGCGAAGC | KdsD gene specific primer |  |
| KdsD_GS_Rev | CGACGACGAGCATCTGATTG | KdsD gene specific primer |  |
| KdsD_SP_For | AGCGACATCGCGAGATCCTTCGAC | KdsD mutant screening primer |  |
| KdsD_SP_Rev | CGCGAAATAGTCGGGCGTGTG | KdsD mutant screening primer |  |
| Ec_KdsD_For | AGAAGATCTATGTCGCACGTAGAGTTACAAC | Expression of recombinant E.coli KdsD | BglII |
| Ec_KdsD_Rev | ACTAAGCTTTTACACTACGCCTGCACGCAG | Expression of recombinant E.coli KdsD | HindIII |
| Bps_KdsD_For | AGAAGATCTATGATAGCGAAAATCAATGAT | Expression of recombinant B. pseudomallei KdsD | BglII |
| Bps_KdsD_Rev | ACTAAGCTTTCAAATCACCTTCTTCGAGAA | Expression of recombinant B. pseudomallei KdsD | HindIII |
